# Neck dissection does not increases the risk of stroke in thyroid cancer: A national cohort study

**DOI:** 10.1371/journal.pone.0195074

**Published:** 2018-03-29

**Authors:** Bumjung Park, Chanyang Min, Hyo Geun Choi

**Affiliations:** 1 Department of Otorhinolaryngology-Head & Neck Surgery, Hallym University College of Medicine, Anyang, Korea; 2 Hallym Data Science Laboratory, Hallym University College of Medicine, Anyang, Korea; University of South Alabama Mitchell Cancer Institute, UNITED STATES

## Abstract

**Objectives:**

The purpose of this study is to evaluate the risk of stroke (hemorrhagic or ischemic) after neck dissection in thyroid cancer patients in Korea using national cohort data.

**Methods:**

Using the national cohort study from the Korean Health Insurance Review and Assessment Service, patients with neck dissection for thyroid cancer (1,041) and control participants (4,164) were selected and matched 1:4 (age, gender, income, and region of residence). The Chi-square test, Fischer’s exact test, and the Cox-proportional hazard model were used. The Cox-proportional analysis used a crude model and an adjusted model for age, gender, income, region of residence, hypertension, diabetes, dyslipidemia.

**Results:**

None of the participants had suffered hemorrhagic stroke in the neck dissection group, while 0.3% (13/4,164) of participants had suffered hemorrhagic stroke in the control group (P = 0.085). In total, 0.8% (8/1,041) of participants had suffered an ischemic stroke in the neck dissection group, and 0.7% (31/4,133) of participants had suffered an ischemic stroke in the control group (P = 0.936). The adjusted hazard ratio for ischemic stroke after neck dissection was 1.06 (95% confidence interval [CI] = 0.49–2.31, P = 0.884).

**Conclusion:**

The risk of hemorrhagic or ischemic stroke was not higher in thyroid cancer patients who underwent neck dissection than that in the matched control group.

## Introduction

Neck dissection is a common treatment option for lymph node metastasis in patients with head and neck cancer. A high risk of stroke was previously reported to exist after neck dissection [1,2]. Nosan et al. reported that 5 of 105 patients (4.9%) had a postoperative stroke [1]. Rechtweg et al. described their experience with neck dissection with simultaneous carotid endarterectomy [2]. In their discussion, Yoo et al. stated that 3.2% of patients among 441 patients with 560 neck dissections suffered a stroke [2]. The neck dissection technique requires the rotation and extension of the neck, which causes intimal tearing of the carotid artery and thrombus formation or plaque ulceration from the turbulent flow [3]. Additionally, neck dissection could result in hemodynamic instability, blood loss, and the exposure and handling of the vascular structure of the neck, which could increase the risk of stroke [4]. However, Thompson et al. reported a low incidence of stroke after neck dissection (0.2%) and concluded that perioperative screening or intervention is not needed [4]. In recent studies, MacNeil et al. reported a low incidence (0.7%) of ischemic stroke in the 30 days after neck dissection, similar to that of thoracic surgery and colectomy [5]. Chang et al. reported that neck dissection does not increase the incidence of stoke in oral cavity cancer patients [6].

Previous studies have evaluated head and neck cancer patients, including patients with oral cavity, nasopharynx, larynx, and hypopharynx cancers [4–6]. However, these studies could not exclude the confounding factors between head and neck cancer and stroke, such as smoking and alcohol use. Increasing age is another confounder [7]. Moreover, the use of radiation and chemotherapy for head and neck cancer could increase the risk of stroke [8]. Therefore, we propose that the risk of neck dissection can be evaluated independently in thyroid cancer patients because smoking and alcohol consumption are not evident risk factors for thyroid cancer [9, 10], and radiation and chemotherapy, apart from radioiodine therapy, are not common treatments for thyroid cancer. Because we could not find any study that reported an increased risk of stroke after radioiodine therapy, we did not evaluate the effect of radioiodine therapy in this study. The purpose of this study is to evaluate the risk of stroke after neck dissection in thyroid cancer patients. We compared the risk of stroke in an age-, gender-, income-, region of residence-, and index date-matched control group that had not been diagnosed with any neoplasm.

## Materials and methods

### Study population and data collection

The ethics committee of Hallym University (2014-I148) approved the use of these data. Written informed consent was exempted by the Institutional Review Board.

This national cohort study relies on data from the Korean Health Insurance Review and Assessment Service—National Patient Sample (HIRA-NPS). The Korean National Health Insurance Service (NHIS) selects samples directly from the entire population database to prevent non-sampling errors. Approximately 2% of the samples (one million) were selected from the entire Korean population (50 million). This selected data can be classified at 1,476 levels (age [18 categories], gender [2 categories], and income level [41 categories]) using randomized stratified systematic sampling methods via proportional allocation to represent the entire population. After data selection, the appropriateness of the sample was verified by a statistician who compared the data from the entire Korean population to the sample data. The details of the methods used to perform these procedures are provided by the National Health Insurance Sharing Service [11]. This cohort database included (i) personal information, (ii) health insurance claim codes (procedures and prescriptions), (iii) diagnostic codes using the International Classification of Disease-10 (ICD-10), (iv) socio-economic data (residence and income), and (v) medical examination data for each participant over a period ranging from 2002 to 2013.

Because all Korean citizens are recognized by a 13-digit resident registration number from birth to death, exact population statistics can be determined using this database. It is mandatory for all Koreans to enroll in the NHIS. All Korean hospitals and clinics use the 13-digit resident registration number to register individual patients in the medical insurance system. Therefore, the risk of overlapping medical records is minimal, even if a patient moves from one place to another. Moreover, all medical treatments in Korea can be tracked without exception with the HIRA system.

### Participant selection

**Fig 1:**
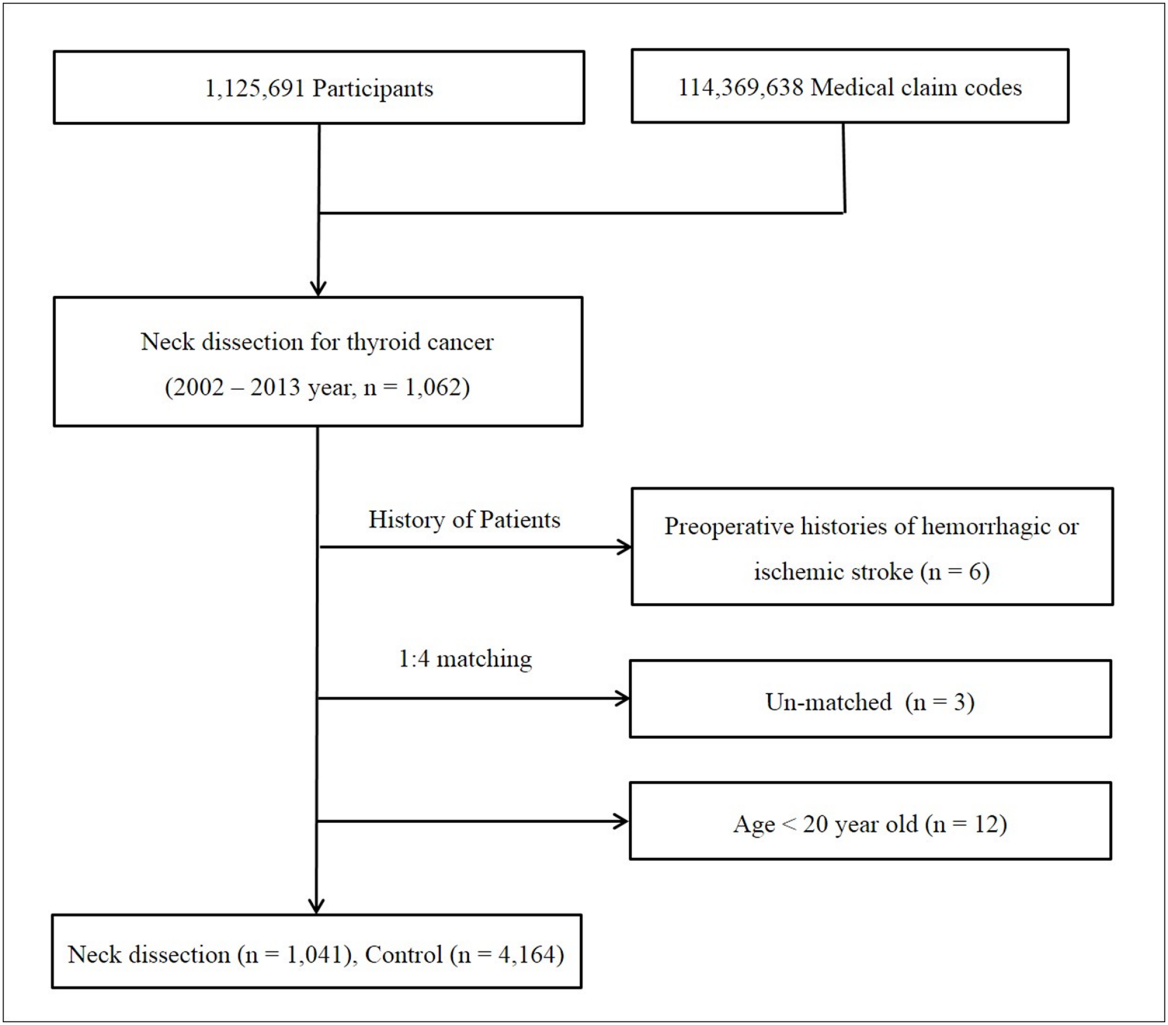
A schematic illustration of the participant selection process that was used in the present study. Of a total of 1,125,691 participants, The neck dissection participants were matched 1:4 with the control group. Finally, 1,041 neck dissection participants and 4,164 control participants were included.

Of the 1,125,691 cases with 114,369,638 medical claim codes, we included participants who underwent modified, radial, or bilateral neck dissection (claim code: P2112, P2113, P2115, P2116, P 2118, and P2119) for thyroid cancer (ICD-10: C73) from 2002 through 2013 (n = 1,062). We did not include participants who underwent selective neck dissection to prevent the analysis of patients who underwent only central neck dissection. Among these patients, participants who had an admission history of hemorrhagic (I60: Subarachnoid haemorrhage, I61: Intracerebral haemorrhage, and I62: Other nontraumatic intracranial haemorrhage) or ischemic stroke (I63: Cerebral infarction) before neck dissection were excluded (n = 6). None of the neck dissection participants were treated with radiation or chemotherapy. The control participants were extracted from mother population. The neck dissection participants were matched 1:4 with the participants (control group) who had never been diagnosed with any neoplasm (C00-D49). Therefore, we excluded any participants who had undergone radiation, chemotherapy, or neck biopsy, which might affect the risk of stroke in the control group. The matches were processed for age, group, gender, income group, region of residence, hypertension, diabetes, dyslipidemia histories, and index date. To prevent selection bias when selecting the matched participants, the control group participants were sorted using random numbers and then selected in order. We assumed that the matched control participants were examined at the same time (index date = the date of neck dissection) as each matched neck dissection participant. Participants with a history of hemorrhagic or ischemic stroke before the index date were excluded from the control group. The neck dissection participants for whom we could not identify enough matching participants were excluded (n = 3). We exclude the participants who are under 20 years old in index date (n = 12). Finally, 1:4 matching resulted in the inclusion of 1,041 of neck dissection participants and 4,164 control participants (Fig 1). They were followed up to 12 years.

### Variables

The age groups were classified using 5-year intervals: 20–24, 25–29, 30–34,…, and 85+ years old. A total of 14 age groups were designated. The income groups were initially divided into 41 classes (one health aid class, 20 self-employment health insurance classes, and 20 employment health insurance classes). These groups were re-categorized into 11 classes (class 1 [lowest income]-11 [highest income]). The region of residence was divided into 16 areas according to administrative district. These regions were regrouped into urban (Seoul, Busan, Daegu, Incheon, Gwangju, Daejeon, and Ulsan) and rural (Gyeonggi, Gangwon, Chungcheongbuk, Chungcheongnam, Jeollabuk, Jeollanam, Gyeongsangbuk, Gyeongsangnam, and Jeju) areas.

The past medical histories of participants were evaluated using ICD-10 codes. To establish an accurate record of diagnosis, participants were considered to have hypertension (I10 and I15), diabetes (E10-E49), or dyslipidemia (E78) if they were treated for these conditions ≥ 2 times.

### Statistical analyses

The Chi-square test or Fischer’s exact test were used to compare age, gender, income, region of residence, and the presence of hypertension, diabetes, dyslipidemia, and hemorrhagic and ischemic stroke between the neck dissection and control group. Kaplan-Meier analysis was used to determine the cumulative probability of ischemic stroke after neck dissection. The Cox-proportional hazard model was used to analyze the hazard ratio of ischemic stroke after neck dissection. A crude model and an adjusted model for age, gender, income, region of residence, hypertension, diabetes, and dyslipidemia, were used in this analysis, and the 95% confidence interval (CI) was calculated. We analyzed the participants according to follow up periods (within 1 year, and 3 years). Two-tailed analyses were conducted, and P values less than 0.05 were considered to indicate significance. The results were statistically analyzed with SPSS v. 21.0 (IBM, Armonk, NY, USA).

## Results

The participants in the neck dissection group were exactly matched 1:4 for age, gender, income, residence. Hypertension, diabetes, and dyslipidemia histories; therefore, these variables were the same in the neck dissection and control groups (P = 1.000, Table 1).

**Table 1:** General characteristics of participants.

Characteristics	The Number of participants		
	Neck dissection group	Control group	P-value*
Age (years old) (n, %)			1.000
20–24	11 (1.1)	44 (1.1)	
25–29	44 (4.2)	176 (4.2)	
30–34	82 (7.9)	328 (7.9)	
35–39	117 (11.2)	468 (11.2)	
40–44	137 (13.2)	548 (13.2)	
45–49	150 (14.4)	600 (14.4)	
50–54	171 (16.4)	684 (16.4)	
55–59	123 (11.8)	492 (11.8)	
60–64	94 (9.0)	376 (9.0)	
65–69	56 (5.4)	224 (5.4)	
70–74	41 (3.9)	164 (3.9)	
75–79	10 (1.0)	40 (1.0)	
80–84	4 (0.4)	16 (0.4)	
85+	1 (0.1)	4 (0.1)	
Sex (n, %)			1.000
Male	204 (19.6)	816 (19.6)	
Female	837 (80.4)	3,348 (80.4)	
Income (n, %)			1.000
1 (lowest)	13 (1.2)	52 (1.2)	
2	69 (6.6)	276 (6.6)	
3	59 (5.7)	236 (5.7)	
4	55 (5.3)	220 (5.3)	
5	71 (6.8)	284 (6.8)	
6	82 (7.9)	328 (7.9)	
7	91 (8.7)	364 (8.7)	
8	92 (8.8)	368 (8.8)	
9	127 (12.2)	508 (12.2)	
10	175 (16.8)	700 (16.8)	
11 (highest)	207 (19.9)	828 (19.9)	
Region of residence (n, %)			1.000
Urban	516 (49.6)	2,064 (49.6)	
Rural	525 (50.4)	2,100 (50.4)	
Hypertension (n, %)			1.000
Yes	331 (31.8)	1,324 (31.8)	
No	710 (68.2)	2,840 (68.2)	
Diabetes (n, %)			1.000
Yes	167 (16.0)	668 (16.0)	
No	874 (84.0)	3,496 (84.0)	
Dyslipidemia (n, %)			1.000
Yes	291 (28.0)	1,164 (28.0)	
No	750 (72.0)	3,000 (72.0)	

* Chi-square or Fisher’s exact test

**Fig 2:**
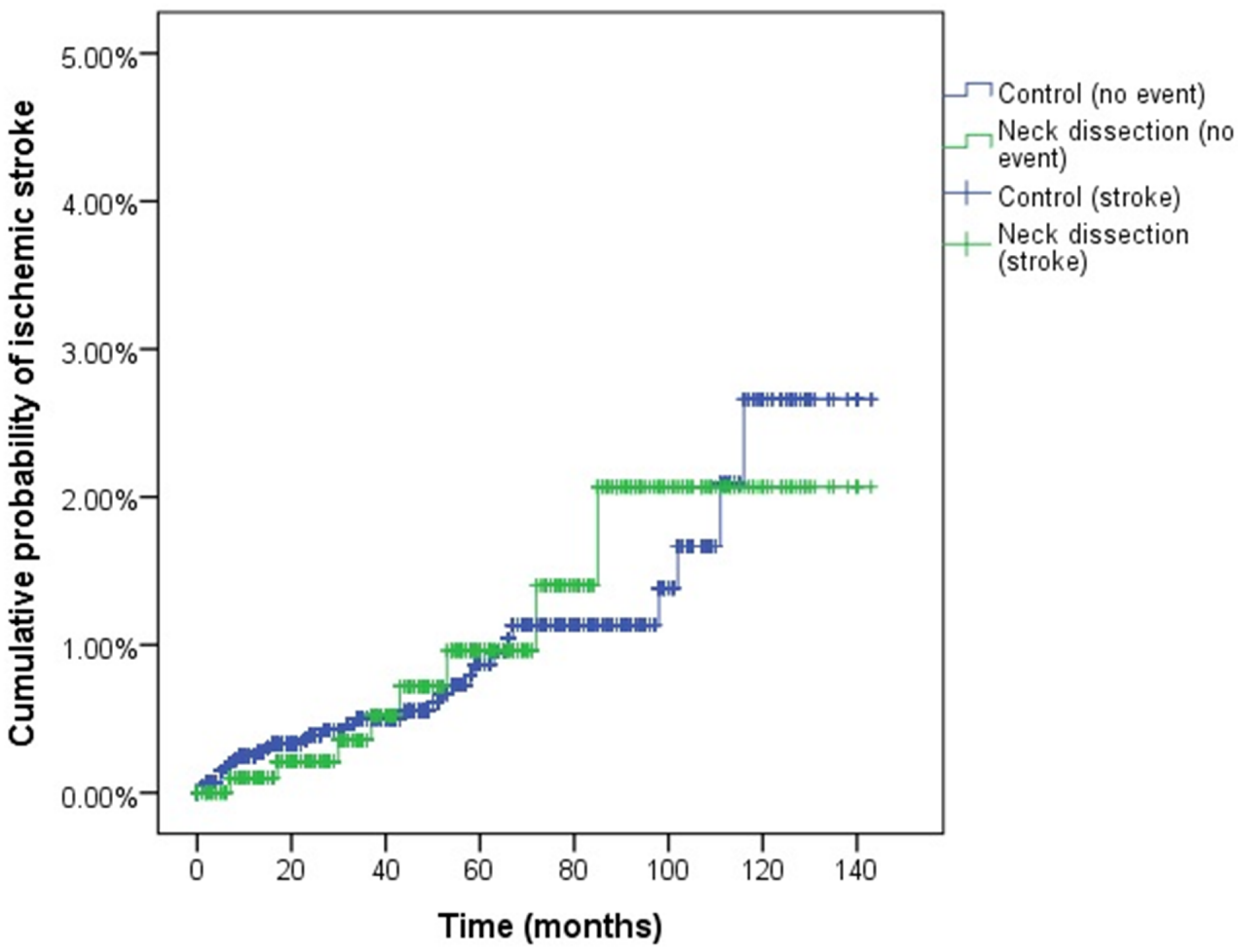
The cumulative probability of ischemic stroke in the neck dissection and control group.

None of the participants had suffered a hemorrhagic stroke in the neck dissection group, while 0.3% (13/4,164) of participants in the control group had experienced a hemorrhagic stroke (P = 0.085). In total, 0.8% (8/1,041) of participants had suffered an ischemic stroke in the neck dissection group, and 0.7% (31/4,133) of participants had suffered an ischemic stroke in the control group (P = 0.936) (Table 2). In the Cox-proportional hazard model, the crude hazard ratio of ischemic stroke was 1.03 (95% CI = 0.48–2.25, P = 0.932), and the adjusted hazard ratio was 1.06 (95% CI = 0.49–2.31, P = 0.884) (Table 3). The cumulative probability of ischemic stroke was not different between the neck dissection group and the control group (Log rank test: P = 0.932, Fig 2). In subgroup analysis according to follow up periods, the crude hazard were not statistically significant within 1 year, and 3 years follow up periods (each P > 0.05, S1 Table).

**Table 2:** The rate of hemorrhage and ischemic stroke after neck dissection for thyroid cancer.

	Neck dissection group (n, %)	Control group (n, %)	P-value*
Hemorrhagic stroke			0.085
Yes	0 (0.0)	13 (0.3)	
No	1,041 (100)	4,151 (99.7)	
Ischemic stroke			0.936
Yes	8 (0.8)	31 (0.7)	
No	1,033 (99.2)	4,133 (99.3)	

* Chi-square or Fisher’s exact test

**Table 3:** Crude and adjusted hazard ratios (95% confidence interval) of neck dissection for ischemic stroke.

Characteristics	Ischemic stroke			
	Crude	P-value*	Adjusted†	P-value*
Neck dissection	1.03 (0.48–2.25)	0.932	1.06 (0.49–2.31)	0.884
Control	1.00		1.00	

* Cox-proportional hazard regression model

^†^ Adjusted model for age, sex, income, region of residence, hypertension, diabetes, dyslipidemia

## Discussion

The risk of hemorrhagic or ischemic stroke was not higher in the neck dissection group than that in the matched control group. Previous studies [1, 2] that have reported a higher risk of stroke in patients who underwent neck dissection were performed on a limited number of participants who may have been compared with an inappropriate control group. Our study complements the limitations of recent studies and revealed no increase in the incidence of stroke after neck dissection for head and neck cancer [4–6]. Smoking and excessive alcohol intake are common risk factors for head and neck cancer and stroke [8], but these factors do not affect thyroid cancer [9, 10]. Therefore, the participants who underwent neck dissection for thyroid cancer might not have higher rates of smoking or alcohol intake than the control group. Although older age could be a confounder for thyroid cancer with neck dissection and stroke [7], we matched the participants with regard to age. Therefore, this study could evaluate the separate risk of neck dissection on stroke. In a previous study [8], the risk of stoke was higher in a young age group that received radiation or chemotherapy for head and neck cancer. Because only thyroid cancer patients who had undergone neck dissection were included, the confounding effects of chemotherapy or radiation did not distort this result.

Even though the position of neck dissection and the extension and rotation of the neck could affect the carotid artery during the operation [3], these factors might not be adequate to cause vessel injuries. The manipulation of vessels might not be adequate to injure the endothelial cells of the carotid artery, which could result in thrombosis or plaque formation from the turbulent flow [3]. Previously, the preoperative carotid artery stenosis was suggested as the risk factor for perioperative stroke [12]. In that study, male, elderly, and smoker showed the high rates of carotid artery stenosis over 70%. However, we included the thyroid cancer participant, 80.4% of participants were women, and 80.2% of participant were under 60 years old when they had the neck dissection. Therefore, these differences of general characteristics of participants might affected results.

One advantage of this study is the large number of study participants (n = 5,205). We evaluated the risk of stroke in patients undergoing neck dissection for thyroid cancer and excluded the confounding effects of smoking, alcohol use, radiation therapy or chemotherapy. We included only participants with modified, radial, or bilateral neck dissection. These procedures were radical surgeries that involved manipulation around the carotid artery. Therefore, the possibility that the low risk of stroke was due to minimal neck manipulation is low. We matched the participants according to the presence of hypertension, diabetes, and dyslipidemia that may affect the risk of stroke. We evaluated the medical records of HIRA. Therefore, the no possibility exists that recall bias could have affected the surveyed study. Moreover, the operation codes for claim data are very reliable. The HIRA data include all citizens of the nation, without exception. Therefore, no participants were missing during any of the follow up periods. We matched the control group according to age, gender, income, and region of residence. Our study results are therefore representative of the entire Korean population because the data were selected from a database that covers the entire population, and the representativeness of the data was verified by a statistician.

Our study has several limitations. Despite the relatively large number of participants, none of the participants in the neck dissection stroke had suffered a hemorrhagic stroke due to its low incidence. Because we examined the medical records of participants from 2002 through 2013, patients who underwent neck dissection, radiation therapy, or chemotherapy before 2002 may have been included in the control group. Even though we used the medical claim codes of stroke like the same way of the previous studies that reported the incidence of stoke of Korea [13, 14], it does not guarantee the exact diagnosis of stroke. Because none of the participants revealed the hemorrhagic or ischemic stroke event within 6 months after neck dissection in this study, we could not evaluate the effects of neck dissection on stroke in the short time periods.

## Conclusion

The risk of hemorrhagic or ischemic stroke was not higher after neck dissection in thyroid cancer participants than that in the matched control group.

## Supporting information

S1 Table Subgroup analysis of the crude ratios of ischemic stroke after neck dissection according the follow up period. (DOCX)
