# Supplementary material for: Neck dissection does not increases the risk of stroke in thyroid cancer: A national cohort study
Source: PLoS One. 2018 Mar 29;13(3):e0195074. doi: 10.1371/journal.pone.0195074 (PMC5875838; doi:10.1371/journal.pone.0195074)
Supplement: S1 Table — (DOCX) [file pone.0195074.s001.docx]

**Supplement 1** Crude hazard ratios (95% confidence interval) of neck dissection for ischemic stroke according to follow up periods

| Characteristics | | Ischemic stroke | |
| --- | --- | --- | --- |
|  |  | Crude | P-value |
| Within 1 year | |  |  |
|  | Neck dissection | 0.40 (0.05-3.12) | 0.382 |
|  | Control | 1.00 |  |
| Within 3 years | |  |  |
|  | Neck dissection | 0.66 (0.20-2.26) | 0.514 |
|  | Control | 1.00 |  |

* Cox-proportional hazard regression model, Significance at P < 0.05
